# The Ameliorative Role of Eugenol against Silver Nanoparticles-Induced Hepatotoxicity in Male Wistar Rats

**DOI:** 10.1155/2022/3820848

**Published:** 2022-09-10

**Authors:** Hany N. Yousef, Somaya S. Ibraheim, Ramadan A. Ramadan, Hanaa R. Aboelwafa

**Affiliations:** Department of Biological and Geological Sciences, Faculty of Education, Ain Shams University, Cairo 11566, Egypt

## Abstract

**Background:**

Silver nanoparticles (AgNPs) utilization is becoming increasingly popular. The existing investigation evaluates the ameliorative impact of eugenol (Eug) against the toxic influences of AgNPs on rats' liver.

**Methods:**

Sixty adult male rats were enrolled equally into control, Eug (100 mg kg^−1^ orally), AgNPs-low dose (1 mg kg^−1^ i.p), AgNPs-high dose (2 mg kg^−1^ i.p), Eug + AgNPs-low dose (100 mg kg^−1^ orally + 1 mg kg^−1^ i.p), and Eug + AgNPs high dose (100 mg kg^−1^ orally + 2 mg kg^−1^ i.p). All the groups were treated daily for 30 days, subsequently serum aspartate transaminase (AST), alanine transaminase (ALT), alkaline phosphatase (ALP), total protein, total albumin, lactate dehydrogenase (LDH), total oxidative capacity (TOC), malondialdehyde (MDA), tumor necrosis factor-alpha (TNF-*α*), total antioxidant capacity (TAC), and interleukin 6 (IL-6) levels were measured; hepatic tissues superoxide dismutase (SOD), catalase (CAT), reduced glutathione (GSH), and glutathione peroxidase (GPx) levels were evaluated; histopathology and histomorphometry were documented in the liver of all groups; and Bcl-2, P53, Caspase-3, and TNF-*α* reactive proteins were also immunohistochemically detected.

**Results:**

AgNPs significantly triggered oxidative stress in hepatic tissues, characterized by elevated levels of AST, ALT, ALP, LDH, TOC, MDA, TNF-*α*, and IL-6 correlating with considerable decline in total protein, total albumin, TAC, SOD, CAT, GSH, and GPx. These changes were paralleled with histopathological alterations remarkable by devastation of the ordinary hepatic structure, with decrease in the numbers of normal hepatocytes, elevation in the numbers of necrotic hepatocytes, periportal and centrilobular inflammatory cells, deteriorated Kupffer cells, and dilated/congested central and portal veins. Alongside, a marked diminution in Bcl-2 immunoreactivity and a significant elevation in P53, Caspase-3, and TNF-*α* immunoreactivities were recorded. Supplementation of AgNPs-treated animals with Eug reversed most of the biochemical, histopathological, and immunohistochemical changes.

**Conclusion:**

This study proposed that Eug has an ameliorative effect against AgNPs-induced hepatotoxicity.

## 1. Introduction

Nanoparticles (NPs) are materials with a particle magnitude ranging from 1 to 100 nm that may be of natural inception or can be created by human industrial activities. Owing to their size, surface charge, and surface area, NPs possess unique physicochemical characteristics [1]. There is an expanding demand for using NPs in drug and medical services, materials science, industrial and household applications, electronics, energy harvesting, and mechanical industries [2].

Silver nanoparticles (AgNPs) attracted immense research attention worldwide consequent to their eminent physical, chemical, and biological advantages. AgNPs are utilized as antimicrobial substances in many industries like biomedicine [3], cosmetics [4], food packaging [5], textile coating [6], and water disinfection [7]. AgNPs are also employed in many other fields, such as molecular imaging [8], drug delivery, and anticancer therapeutics [9].

The expanding utilization of nanomaterials in our daily life increases environmental worries. AgNPs can either purposely or unpurposely enter the human body via inhalation, drinking, ingestion, skin uptake, or intravenous injection [2]. Inside the body, AgNPs can translocate and accumulate in different tissues, including the liver, heart, lungs, kidneys, and nervous tissues, where they interfere with the metabolic pathways inside the cells and exert adverse effects and toxicity [10]. The liver is a prime accumulation site of circulatory AgNPs, which cause cellular injury and toxic effects on it [11].

The mechanisms underlying AgNPs-induced cytotoxicity and genotoxicity are unclear. The liberation of silver ions and formation of reactive oxygen species (ROS) are proposed to be the possible reasons for the toxic impacts of AgNPs [12]. The increased intracellular ROS levels induce oxidative stress and subsequent lipid peroxidation and cellular macromolecular damage, which eventually leads to cell death [13].

Numerous investigations have emphasized the antioxidant potential of phytochemicals [14]. Eugenol (Eug; 1-allyl-4-hydroxy-3-methoxybenzene [C_10_H_12_O_2_]) is a phenolic phytochemical abundantly present in clove, cinnamon, and basil and is used as a flavoring substance in foods and cosmetics and as a preservative in foods [15]. Eug has several pharmacological activities, including antimicrobial [16], antioxidant [17], anti-inflammatory [18], and anticancer [19] efficiencies.

According to the current literature, very rare and nonspecific studies on the protective impact of Eug in reducing AgNPs-induced hepatotoxicity in male rats have been done. Thus, the present study's objective was to elucidate Eug's potential ameliorative effect against AgNPs-induced hepatotoxicity in male rats employing biochemical, histological, histomorphometrical, and immunohistochemical approaches.

## 2. Materials and Methods

### 2.1. Chemicals Used

Silver nitrate (AgNO_3_, 99%), gelatin, and eugenol (Eug, 99%) were obtained from Sigma-Aldrich (St. Louis, MO, USA). Additional reagents and chemicals used were of high analytical grade and pureness.

### 2.2. Synthesis of AgNPs

AgNPs were synthesized using AgNO_3_ and gelatin via the microwave method according to [20]. 16.987 g of AgNO_3_ was dissolved in 100 mL of deionized distilled water for 20 min. Next, 1 g of gelatin soluble in deionized distilled water was added to AgNPs. The solution was stirred at 60°C for 24 h on a direct hot plate and then subjected to microwave irradiation at 700 watts for 5 min. All samples were naturally chilled at room temperature, and carefully covered and capped.

### 2.3. Physicochemical Characterization of AgNPs

Ultraviolet-visible (UV-VIS) spectroscopy (JASCO V-550 UV/VIS double-beam spectrophotometer, Tokyo, Japan) was used for the primary characterization of the synthesized AgNPs and to monitor their synthesis and stability. The crystallinity of AgNPs was characterized using an X-ray diffractometer (XRD) employing CuK*α* radiation. The sample was scanned over a 2Ɵ range of 10°-80°. The chemical state and elemental composition were measured via X-ray photoelectron spectroscopy (XPS). The morphology of NPs was investigated by scanning electron microscopy (SEM) operating at 20 kV. Transmission electron microscopy (TEM) was performed to monitor the size and morphological stability of AgNPs [21]. AgNPs samples from the stock suspensions were sonicated in nanopure water for 30 min at room temperature. Then, a drop from the dilute specimen solution was dropped onto an amorphous carbon-coated copper grid and air dried, generating a monolayer. The particle diameters of the synthesized AgNPs were assessed using the software image analysis program over various shots of TEM photomicrographs for the intended specimen [22] at the Central Laboratory, Faculty of Agriculture, Cairo University, Egypt.

### 2.4. Experimental Animals

Sixty males of albino rats (*Rattus norvegicus*) of nearly equal age and weighing 180-200 g were obtained from the Schistosoma Biological Supply Program, Theodor Bilharz Research Institute, El-Giza, Egypt. The rats were kept in clean plastic crates supplied with wood shavings and fed a regular rodent pellet diet, besides water *ad libitum* at room temperature (25 ± 2°C), with a 12/12-h light-dark period and 55 ± 5% relative humidity. Before experimentation, all rats were allowed to adapt for a week. This study was in compliance with the international standards for animal laboratory treatment set by the local Institutional Animal Ethics Committee of Ain Shams University for the use and treatment of animals.

### 2.5. Experimental Design

The rats were put into six groups, each with 10 animals. They were treated daily at 9 a.m. for 30 days as follows:

Control group: healthy rats intraperitoneally (i.p.) injected with 1 mL deionized distilled water (vehicle for AgNPs) and orally received 1 mL corn oil (vehicle for Eug) by oral gavage.

Eug-treated group: rats were orally given Eug (100 mg kg^−1^ body weight) suspended in 1 mL of corn oil by oral gavage. This dose was calculated according to the dose used in previous rat studies [23].

AgNPs-low-dose-treated group: rats were i.p. treated with AgNPs (1 mg kg^−1^ body weight) dissolved in 1 mL of deionized distilled water.

AgNPs-high-dose-treated group: rats were i.p. treated with AgNPs (2 mg kg^−1^ body weight) dissolved in 1 mL of deionized distilled water.

The low and high doses of AgNPs were selected based on those used in previous investigations [2].

Eug + AgNPs-low-dose-treated group: rats were orally given Eug (100 mg kg^−1^ body weight) paralleled with i.p. injection of AgNPs-low dose (1 mg kg^−1^ body weight).

Eug + AgNPs-high-dose-treated group: rats were orally given Eug (100 mg kg^−1^ body weight) paralleled with i.p. injection of AgNPs-high dose (2 mg kg^−1^ body weight).

### 2.6. Collection of Sera and Tissue Samples

At the end of all treatments, the rats were fasted nightly, and in the following morning, they were anesthetized with light ether anesthesia. Using cardiac puncture, samples of blood were obtained and centrifuged for 10 minutes at 1500 × g and 4°C to get sera that were promptly preserved at −80°C until usage. The livers of dissected rats were segregated out and instantly rinsed with ice-cold 0.9% NaCl physiological saline, and then they were kept frozen at −80°C for additional biochemical investigations, while the other liver specimens were excised and prepared for the histological, histomorphometrical, and immunohistochemical studies.

### 2.7. Preparation of Liver Homogenates

Using Ultra Turrax tissue homogenizer, specimens from the liver were homogenized in pH 7.4 ice-cold phosphate-buffered saline (PBS) to obtain a 10% solution (w/v). 0.16 mg/mL heparin was added to PBS to eliminate any erythrocytes and clots. The obtained homogenate was centrifuged for 15 min at 9000 × g and 4°C, and then the clear supernatant was separated and preserved frozen at -80°C for subsequent biochemical assays. Hepatic protein content in every sample was determined using the previously reported procedures [24] which used bovine serum albumin as a standard.

### 2.8. Biochemical Assessment

#### 2.8.1. Liver Function Biomarkers

Colorimetric assay kits specific for liver function biomarkers (aspartate aminotransferase (AST), alanine aminotransferase (ALT), alkaline phosphatase (ALP), lactate dehydrogenase (LDH), total protein, and total albumin) were purchased from EGY-CHEM for lab technology (BioMed, Germany) and used in conjunction with UV-VIS spectrophotometer (Shimadzu, Kyoto, Japan). Measuring the activities of AST, ALT, and ALP were based on protocols previously described by [25], while activity of LDH was depended on the protocol of [26]. Serum total protein and albumin levels were determined using the methods outlined by [27, 28], respectively.

#### 2.8.2. Oxidative Stress Biomarkers in Sera

Total antioxidant capacity (TAC) and total oxidant capacity (TOC) were estimated in sera utilizing colorimetric assay kits manufactured by Biomedica Medizinprodukte GmbH, Germany, following the procedures formerly described by [29, 30], respectively.

#### 2.8.3. Oxidative Stress Biomarkers in Liver Tissues

Levels of lipid peroxidation (LPO) were assessed depending on the production of thiobarbituric acid reactive substances (TBARS) and expressed as the extent of malondialdehyde (MDA) formation using a colorimetric assay kit (Biodiagnostic, Egypt) according to [31].

The effectiveness of antioxidants in the liver tissues including superoxide dismutase (SOD), catalase (CAT), reduced glutathione (GSH), and glutathione peroxidase (GPx) were assayed using commercially accessible colorimetric kits (Biodiagnostic, Egypt) utilizing a UV-VIS spectrophotometer (Shimadzu, Kyoto, Japan). The protocol previously published by [32] was applied to estimate the effectiveness of SOD, whereas CAT activity was assayed by H_2_O_2_ consumption according to the method proposed by [33], while the techniques previously depicted by [34, 35] were used for measuring the activities of GSH and GPx, respectively.

#### 2.8.4. Proinflammatory Markers in Liver Tissues

Two proinflammatory cytokines, tumor necrosis factor-alpha (TNF-*α*) and interleukin 6 (IL-6), had been assessed in the liver tissues. TNF-*α* levels were measured using a commercially accessible enzyme-linked immunosorbent assay (ELISA) kits (catalog number: CSB-E11987r, Cusabio Biotech Co., Ltd.) following the manufacturer's protocol. Meanwhile, the concentration of IL-6 had been assessed using the Rat IL-6 Quantikine ELISA Kit subsequent to the instructions described by the manufacturer (R&D Systems, Inc., Minneapolis, USA).

### 2.9. Histological Preparation

Samples of the liver of both control and treated animals were cut into tiny portions that instantly fixed for 24 h in 10% buffered formalin solution; after that they were proceeded for the routine protocol of paraffin sectioning previously described by [36]. 4-6-*μ*m-thick paraffin sections were stained with Ehrlich's hematoxylin and eosin (H&E), dehydrated in a set of graded ethyl alcohol concentrations, cleared in xylene, mounted in DPX, and examined and photographed using compound light microscope (Olympus CX 31) provided with a Panasonic CD-220 camera.

### 2.10. Histomorphometrical Estimation

Six random fields from H&E-stained liver sections of all animal groups were selected and histomorphometrically analyzed using a computed image analysis system (Leica QWin, 500 Software, Germany) in the Department of Oral and Dental Pathology, Faculty of Dental Medicine, Al-Azhar University. The numbers of normal hepatocytes, necrotic hepatocytes, deteriorated Kupffer cells, and infiltrating inflammatory cells in the periportal areas (portal inflammation) and in the centrilobular zones (centrilobular inflammation) were estimated. In addition, the numbers of dilated/congested central and portal veins were recorded.

### 2.11. Immunohistochemical Preparation

Immunohistochemical localization of Bcl-2, P53, Caspase-3, and TNF-*α* reactive proteins was performed according to the standard Avidin-Biotin Complex (ABC) protocol [37]. 5-*μ*m-thick buffered formalin-fixed and paraffin-embedded hepatic tissue sectors were deparaffinized, rehydrated, and washed for 10 min in PBS. Endogenous peroxidase efficacy was obstructed with 3% hydrogen peroxide. After that, slides were incubated for 1–2 h at room temperature with the convenient dilution of the primary antibodies as represented in Table 1, followed in a refrigerator nightly at 4°C. Subsequently, they were rinsed in PBS, incubated for 10 min with biotinylated goat antipolyvalent, and then incubated for 1 h with ABC. Next, the sections were rinsed in PBS and incubated in diaminobenzidine tetrahydrochloride (pH 7.2) with 10 mL H_2_O_2_ for 7–9 min forward by PBS. Finally, sections were counterstained with Mayer's hematoxylin, dehydrated, cleared, and covered by cover slips. Negative control slides in the absence of primary antibodies were included for each parameter. The antibodies and all reagents were utilized in concurrence with the manufacturer's directives and recommendations.

### 2.12. Image Analysis

The quantification of immunoreactivity of active Bcl-2, P53, Caspase-3, and TNF-*α* proteins was assessed using the image analysis to estimate the percentage of positive immunostained cells related to the number of all cells assessed for each parameter [38]. The cells were regarded as positive if there was cytoplasmic and/or membranous brown coloration. A computational image analysis system (Leica QWin 500, Germany) at the Department of Oral and Dental Pathology, Faculty of Girls' Dental Medicine, Al-Azhar University, was used to process the images. For each parameter, six randomly selected high-power fields (×200) were captured in each slide with a standard measuring frame of an area 11434.9 mm^2^. The image analyzer was initially adjusted automatically to transform the image analyzer program's measurement units (pixels) into real micrometer units. For statistical analysis, mean percentages of immunoreactive regions were calculated for all samples in each group.

### 2.13. Statistical Analysis

The biochemical, histomorphometrical, and immunohistochemical data recorded in the current investigation were tabulated and statistically analyzed. The values of six samples/group for all experimental animal sets were expressed as mean ± standard error of mean (SEM). Statistical differences among rats' groups were estimated using one-way analysis of variance (ANOVA) subsequent by Tukey post hoc test utilizing the IBM SPSS Statistics for Windows, version 22 (IBM Corp., Armonk, N.Y., USA). Statistical significance was considered when a *P* value was less than 0.05.

## 3. Results

### 3.1. Physicochemical Characterization of Prepared AgNPs

#### 3.1.1. UV–VIS Spectroscopy

**Figure 1:**
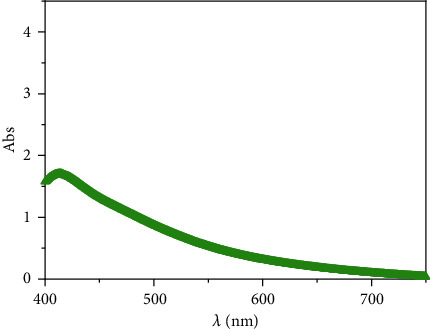
UV–VIS spectrum of AgNPs showing a maximum absorption peak at 413 nm wavelength using 1 g of gelatin via the microwave protocol.

The formation of AgNPs was confirmed by using UV–VIS spectrophotometer. At room temperature, the UV-VIS spectrum of AgNPs showed peak single-band absorption (surface plasmon resonance (SPR)) 1.8 at a wavelength of 413 nm using 1 g of gelatin via the microwave method marked in Figure 1.

#### 3.1.2. X-Ray Diffraction (XRD) Analysis

**Figure 2:**
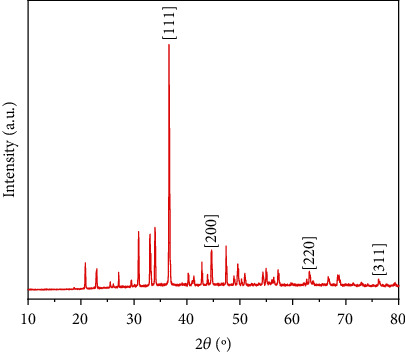
XRD pattern of biosynthesized AgNPs.

The crystal structure of AgNPs was characterized by XRD (Figure 2). The results showed prominent peaks at 2Ɵ = 37.0, 44.6, 63.1, and 76.2, which were assigned to Ag (111), (200), (220), and (311) planes, respectively. These observed planes indicate that AgNPs had a face-centered cube structure and good crystallinity [39, 40]. The observed values agree with the reference of face-centered cubic structure from the Joint Committee of Powder Diffraction Standard (JCPDS No 03-065-2871). The additional peaks detected in the sample were attributed to the presence of AgNO_3_ and AgO.

#### 3.1.3. X-Ray Photoelectron Spectroscopy (XPS) Analysis

**Figure 3:**
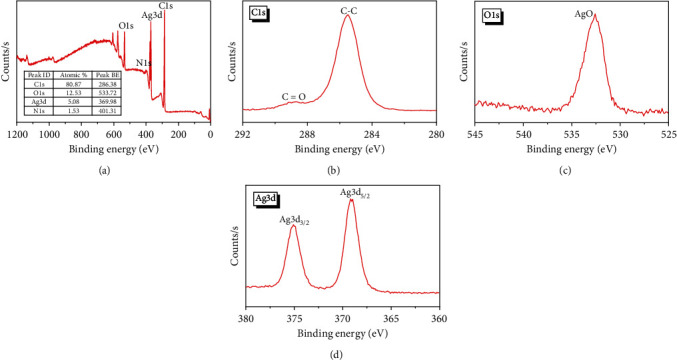
XPS spectra of AgNPs; (a) survey scan and high-resolution spectra of (b) C1s, (c) O1s, and (d) Ag3d.

The XPS results revealed the presence of Ag, C, N, and O atoms according to their binding energies, as shown in Figure 3. There are four peaks in the survey spectra at around 286 eV (C 1 s), 370 eV (Ag 3d), 401 eV (N 1 s), and 534 eV (O 1 s). The elemental compositions determined from XPS analysis were 5.08, 80.87, 1.53, and 12.53 atomic % for Ag, C, N, and O, respectively.

The high-resolution XPS C1s spectrum of the AgNPs showed two peaks at 285.7 and 288.9 eV corresponding to sp^3^ C–C and C=O/C–O–Ag on the AgNPs surface (Figure 3(b)). Moreover, the high binding energy of O 1 s at 532.3 eV was related to metal–OH bonds [41] (Figure 3(c)). The most noticeable signal in the XPS spectrum was that for Ag 3d, which comprises two spin-orbit peaks at 368.7 (Ag 3d_5/2_) and 375.1 (Ag 3d_3/2_) eV separated by around 6.0 eV (Figure 3(d)), which agree with the previously reported results [39, 41]. Together, these results illustrated the successful preparation of AgNPs.

#### 3.1.4. Surface Morphology

**Figure 4:**
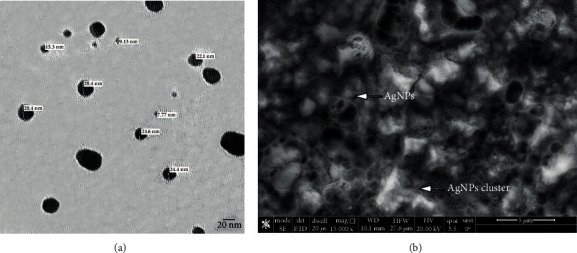
(a) TEM photomicrograph of AgNPs showing spherical forms and excellent particle dispersion with sizes ranging from 7.7 nm to 28.4 nm using 1 g of gelatin by the microwave technique (× 250,000). (b) SEM image showing spherical AgNPs embedded with gelatin obtained at 20 kV electron high tension (EHT).

The morphology of the biosynthesized AgNPs was observed at 20 nm using TEM (Figure 4(a)). TEM analysis showed that the synthesized AgNPs were monodispersed, transparent, spherical, and highly crystalline with a normal small size (7.77-28.4 nm), smooth surface, well-distribution, and no agglomeration and were deemed suitable for utilization in this study. The SEM analysis (Figure 4(b)) of AgNPs showed that the NPs synthesized were spherical in shape [42], which correlated with the observed morphology of TEM image. Some aggregated particles of AgNPs were observed in the prepared sample. The size of the silver particles got larger due to agglomeration of the smaller ones as the bio-organic molecules could stabilize and cap the individual particles [43, 44].

### 3.2. Biochemical Analysis

To evaluate liver injury, liver function enzymes (AST, ALT, ALP, and LDH), total protein, and total albumin levels and oxidant/antioxidant parameters (TAC and TOC) in sera, as well as antioxidant parameters (MDA, SOD, CAT, GSH, and GPX) and inflammation markers (IL-6 and TNF-*α*) in liver tissues, were evaluated.

**Figure 5:**
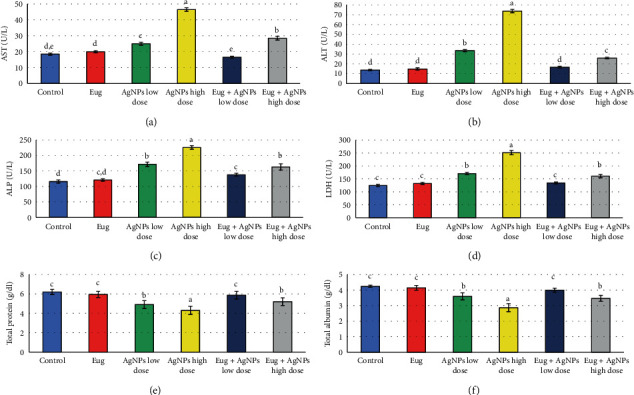
Liver function biomarkers: (a) aspartate aminotransferase (AST), (b) alanine aminotransferase (ALT), (c) alkaline phosphatase (ALP), (d) lactate dehydrogenase (LDH), (e) total protein, and (e) total albumin in the control and treated animal groups. Data are expressed as Mean ± SEM (*n* = 6). Columns with different superscript letters are significantly different at the 0.05 level (one-way ANOVA).

The influences of AgNPs toxicity and the protective effects of Eug on liver function biomarkers of rats are shown in Figure 5. Eug administration alone did not have significant (*P* > 0.05) effects on the measured liver function indices in comparison with the control. Meanwhile, treatment of rats with AgNPs, either the low or high dose, caused marked increase (*P* ≤ 0.05) in AST (35.21% and 151.49%), ALT (145.12% and 439.02%), ALP (48.10% and 94.91%), and LDH (37.14% and 102.31%) paralleled with a significant decline (*P* ≤ 0.05) in total protein (-20.85% and -30.65%) and albumin (-15.66% and -32.47%) compared with the values of control animals. Levels of AST, ALT, LDH, total proteins, and total albumin were restored approximately to the normal levels in rats treated with AgNPs-low dose in combination with Eug. While supplementation of Eug to the rats received the high dose of AgNPs caused modulation of the liver function parameters compared with the animals exposed to the high dose of AgNPs, but they were still considerably different (*P* ≤ 0.05) comparable to the control values.

**Figure 6:**
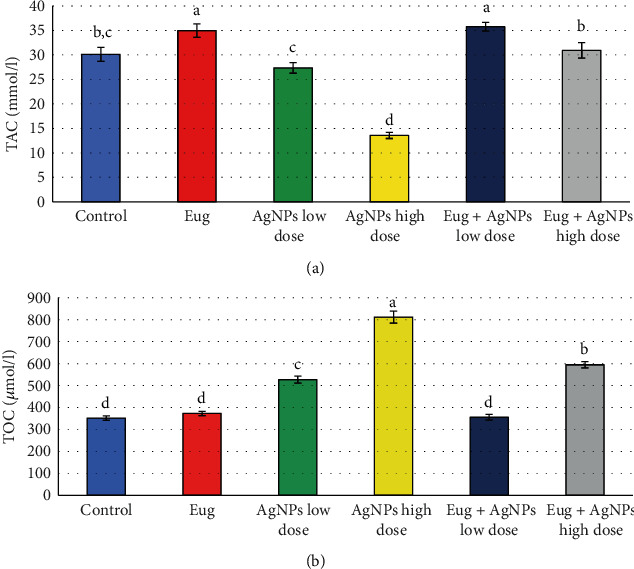
(a) Total antioxidant capacity (TAC) and (b) total oxidant capacity (TOC) in sera of the control and treated animal groups. Data are expressed as mean ± SEM (*n* = 6). Columns with different superscript letters are significantly different at the 0.05 level (one-way ANOVA).

Figure 6 represents the serum TAC and TOC levels in the rats of all groups. The recorded data exhibited a significant rise (*P* ≤ 0.05) in TAC level (16.07%) in Eug-treated group when contrasted with control group. On contrarily, nonsignificant decline (*P* > 0.05) and significant diminution (*P* ≤ 0.05) were recorded in TAC levels of the AgNPs-low-dose-treated rats (-9.19%) and the AgNPs-high-dose-treated rats (-55.01%), respectively, when contrasted with control group. Addition of Eug to the rats injected with AgNPs, both low and high doses, caused modulation of the levels of TAC relative to that recorded in the animals administered AgNPs alone; nevertheless, it was significantly different (*P* ≤ 0.05) compared with the control values in case of the high dose AgNPs-treated rats. Regarding TOC, administration of Eug solely did not produce significant (*P* > 0.05) effects on the levels of this parameter when compared with the control group, while the incredible rise (*P* ≤ 0.05) in levels of TOC of AgNPs-low-dose-treated rats (49.90%) and AgNPs-high-dose-treated rats (130.98%) was recorded when contrasted with the control group, whereas treatment of rats with AgNPs-low dose in combination with Eug returned the values of TOC to be very close to those of the control animals (355.5 ± 12.76 vs. 351.5 ± 10.44, *P* ≥ 0.05), while administration of Eug to the rats treated with the high dose of AgNPs caused amelioration of this parameter when contrasted with the animals treated to AgNPs alone, and this value was yet significantly different (*P* ≤ 0.05) proportional to the control values.

**Figure 7:**
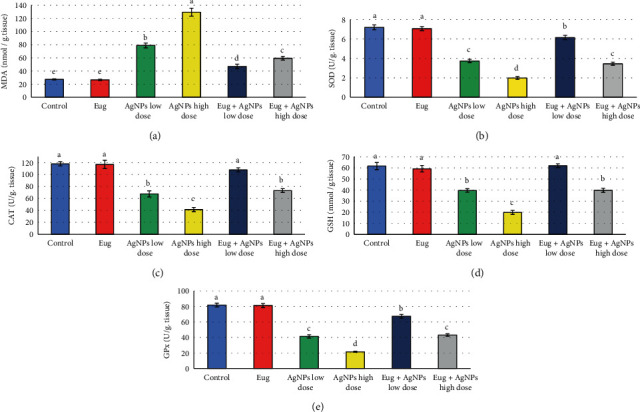
Oxidative stress biomarkers: (a) malondialdehyde (MDA), (b) superoxide dismutase (SOD), (c) catalase (CAT), (d) reduced glutathione (GSH), and (e) glutathione peroxidase (GPx) in the liver tissues of the control and treated animal groups. Data are expressed as mean ± SEM (*n* = 6). Columns with different superscript letters are significantly different at the 0.05 level (one-way ANOVA).

Levels of MDA, SOD, CAT, GSH, and GPx in hepatic tissues of the control and treated animal groups were assessed to monitor the oxidative stress state.  Figure 7 demonstrated that administration of Eug alone did not produce significant (*P* > 0.05) effects on these oxidative stress indices compared with the control. Meanwhile, low- and high-dose AgNPs-treated rats were subjected to oxidative stress which was confirmed by significant elevation (*P* ≤ 0.05) in MDA levels (189.72% and 374.63%) accompanied by marked decline (*P* ≤ 0.05) in levels of SOD (-48.07% and -72.54%), CAT (-42.61% and -64.80%), GSH (-35.63% and -67.69%), and GPx (-49.39% and -73.67%) in comparison with the control group. Eug + AgNPs-low-dose and Eug + AgNPs-high-dose-treated groups showed marked modulation (*P* ≤ 0.05) of the evaluated oxidative stress biomarkers compared with the animals subjected to AgNPs-low dose and AgNPs-high dose; but most of these parameters were significantly different (*P* ≤ 0.05) relative to the recorded control values.

**Figure 8:**
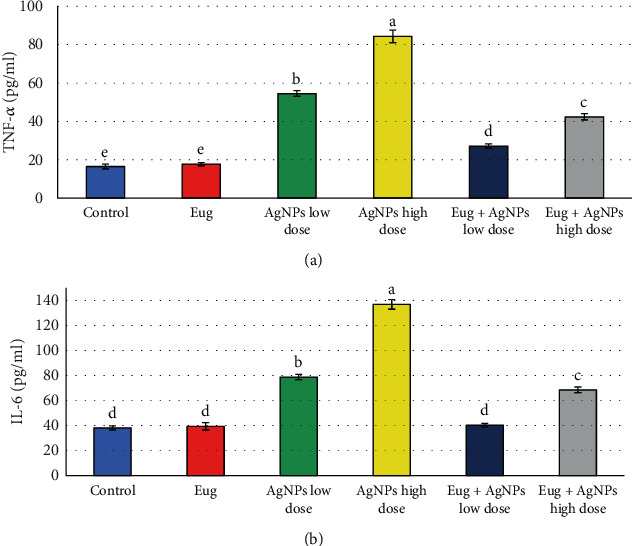
Inflammatory biomarkers: (a) tumor necrosis factor-alpha (TNF-*α*) and (b) interleukin 6 (IL-6) in the liver tissues of the control and treated animal groups. Data are expressed as mean ± SEM (*n* = 6). Columns with different superscript letters are significantly different at the 0.05 level (one-way ANOVA).

Levels of inflammatory biomarkers (TNF-*α* and IL-6) in liver tissues were investigated in all groups, and the within-group values of these parameters were compared (Figure 8). Rats received Eug solely displayed nonsignificant (*P* > 0.05) changes in the levels of TNF-*α* and IL-6 when compared with the control. Meanwhile, treatment of rats with AgNPs resulted in marked elevation (*P* ≤ 0.05) in levels of TNF-*α* (230.07% and 410.31%) and IL-6 (106.40% and 258.36%) for the low dose and high dose of AgNPs, respectively, compared with those of control animals. Administration of Eug along with AgNPs (both low or high doses) significantly ameliorated these changes and recovered to the control levels.

### 3.3. Histological Results

**Figure 9:**
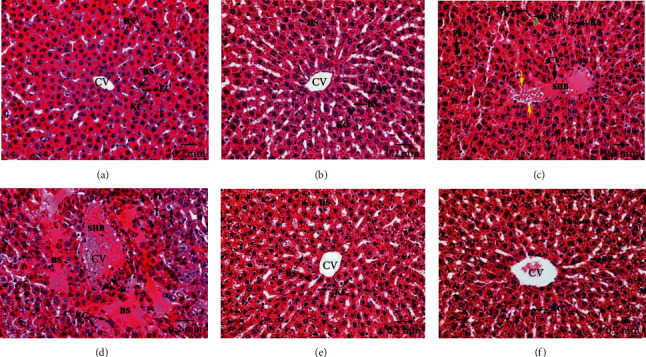
Centrilobular zones of hepatic tissues stained with H&E revealing (a) well-organized hepatic strands (HS) which expanded around a narrowed central vein (CV) and segregated by normal blood sinusoids (BS) appeared enveloped by attached endothelial (EC) and Kupffer (KC) cells in control rats; (b) intact hepatic architecture in Eug-treated rats; (c) dilated and congested central vein (CV) possesses sever stagnant hemorrhagic blood (SHB) in its lumen, and its surrounded endothelium seemed destructed (yellow arrow), and the hepatic blood sinusoids (BS) are dilated and congested with stagnant blood (green asterisk), and their Kupffer cells (KC) appeared more rounded and often pushed into the sinusoidal lumen, as well as hepatocytes showed pyknotic nuclei (Pk), and the others revealed nuclear karyorrhexis (Kh) in AgNPs-low-dose-treated rats; (d) severely dilated and congested central vein (CV) having stagnant blood masses (SHB) and infiltrating inflammatory cells (IC) in its lumen and also aggregated in the surrounding connective tissues, besides enlarged blood sinusoids (BS) with swollen and detached Kupffer cells (KC), disorderly hepatic strands with necrotic hepatocytes having pyknotic nuclei (Pk) and rather vacuolated cytoplasm (V) in AgNPs-high-dose-treated rats; (e) conspicuous amelioration in the structure of hepatic strands (HS), blood sinusoids (BS), and central veins (CV) in Eug + AgNPs-low-dose-treated rats; (f) almost intact architecture of the central vein (CV), blood sinusoids (BS), and hepatocytes with some of them still showing rather nuclear pyknosis (Pk) in Eug + AgNPs-high-dose-treated rats.

**Figure 10:**
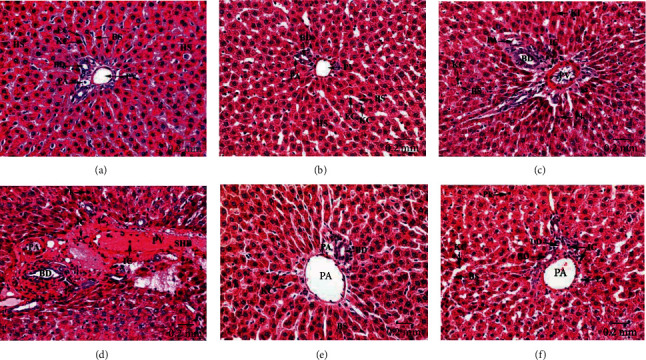
Periportal zones of hepatic tissues stained with H&E revealing (a) regular hepatic architecture with well-organized radiating hepatic strands (HS) surrounding the portal tract that formed of regular hepatic portal artery (PA), hepatic portal vein (PV), and bile ductule (BD) in control rats; (b) regular hepatic structure in Eug-treated rats; (c) devastated hepatic portal artery (PA), hepatic portal vein (PV), and bile ductule (BD) with thickened boundaries, infiltrating inflammatory cells (IC) in the surrounding connective tissues and inside their lumina, besides necrotic hepatocytes with pyknotic (Pk) or karyolysed (KI) nuclei and blood sinusoids (BS) having swollen Kupffer cells (KC) pushed in their lumens are obviously noticed in AgNPs-low-dose-treated rats; (d) severely dilated and congested hepatic portal vein (PV) and portal artery (PA) having stagnant blood masses (SHB) and infiltrating inflammatory cells (IC) in their lumens and also aggregated outside them, besides deteriorated bile ductules (BD) with thickened and deformed epithelia. Also, disorderly hepatic strands with necrotic hepatocytes having pyknotic (PK) nuclei are observed in AgNPs-high-dose-treated rats; (e) conspicuous improvement in the histological structure of the hepatic tissues with intact portal triad and hepatic strands in Eug + AgNPs-low-dose-treated rats; (f) restoration of the hepatic architecture with few infiltrating inflammatory cells (IC) and hepatocytes with pyknotic (Pk) nuclei are noticed in Eug + AgNPs-high-dose-treated rats.

Liver sections of the control and Eug-treated rats revealed ordinary parenchymal architecture of the hepatic tissues in both centrilobular (Figures 9(a) and 9(b)) and the periportal (Figures 10(a) and 10(b)) zones, where well-organized radiating hepatic strands seemed expanded, surrounding the narrowed central veins and also around the portal tracts that formed of the regular hepatic portal veins, hepatic portal arteries, and bile ductules. Besides, normal blood sinusoids lined with attached endothelial and Kupffer cells were observed, while examination of the centrilobular (Figure 9(c)) and the periportal (Figure 10(c)) areas of liver sections of AgNPs-low-dose-treated rats revealed dilated and congested central veins, hepatic portal veins, and hepatic portal arteries which showed thickened and eroded lining membranes surrounded by infiltrating inflammatory cells. Necrotic hepatocytes appeared with nuclear pyknosis, karyorrhexis, and karyolysis. Besides, congested hepatic blood sinusoids with detached endothelial cells and rounded Kupffer cells appeared pushed into the sinusoidal lumen were seen. Moreover, the hepatic tissues of AgNPs-high-dose-treated rats showed massive pathological changes in the centrilobular (Figure 9(d)) and the periportal (Figure 10(d)) zones, where the hepatic cords missed their typical arrangement and the majority of hepatocytes degenerated, having vacuolated cytoplasm and pyknotic, karyorrhectic, or karyolysed nuclei. The central veins, hepatic portal veins, and hepatic portal arteries were disfigured, dilated, and severely congested with intense masses of hemolyzed blood in their lumina, besides infiltration of inflammatory lymphocytes around their confines. In certain areas, the endothelial lining of most of these blood vessels looked corroded and damaged. Furthermore, the blood sinusoids showed destruction and congestion and lined with swollen activated Kupffer cells which appeared separated from their boundaries. Otherwise, Eug + AgNPs-low-dose-treated rats illustrated remarkable refinement in the hepatic architecture of both centrilobular (Figure 9(e)) and the periportal (Figure 10(e)) zones, with most of the hepatocytes and blood sinusoids appeared well-organized. Also, the central and hepatic portal veins, as well as the hepatic portal arteries appeared intact. Similarly, Eug + AgNPs-high-dose-treated rats manifested a nearly well-organized hepatic architecture in the centrilobular (Figure 9(f)) and periportal (Figure 10(f)) zones.

### 3.4. Histomorphometrical Results

As illustrated in Table 2, the hepatic tissues of both AgNPs-low-dose and AgNPs-high-dose-treated rats manifested a significant decline (*P* ≤ 0.05) in the numbers of normal hepatocytes and a considerable rise (*P* ≤ 0.05) in the numbers of necrotic hepatocytes, infiltrating inflammatory cells in the periportal and centrilobular zones, and deteriorated Kupffer cells. In addition, the numbers of dilated/congested central and portal veins were significantly elevated (*P* ≤ 0.05) compared with control and Eug-treated rats. Otherwise, Eug + AgNPs-low-dose- and Eug + AgNPs-high-dose-treated rats showed amelioration of these impaired parameters observed after AgNPs treatment. Necrotic hepatocytes and infiltrating inflammatory cells significantly (*P* ≤ 0.05) decreased in the centrilobular and periportal zones of the hepatic tissues of Eug + AgNPs-low-dose- and Eug + AgNPs-high-dose-treated rats. Meanwhile, a significant (*P* ≤ 0.05) elevation in the numbers of deteriorated Kupffer cells, and dilated/congested central and portal veins, with a marked (*P* ≤ 0.05) depletion in the number of normal hepatocytes were recorded in Eug + AgNPs-low-dose- and Eug + AgNPs-high-dose-treated rats relative to the control rats but showed modulation of these parameters compared with rats treated with AgNPs alone. Eug-treated rats did not show any significant different in these parameters, except for the number of necrotic hepatocytes and infiltrating inflammatory cells, indicating a significant change (*P* ≤ 0.05) from all other groups.

### 3.5. Immunohistochemical Results

#### 3.5.1. Bcl-2 Immunoreactivity

**Figure 11:**
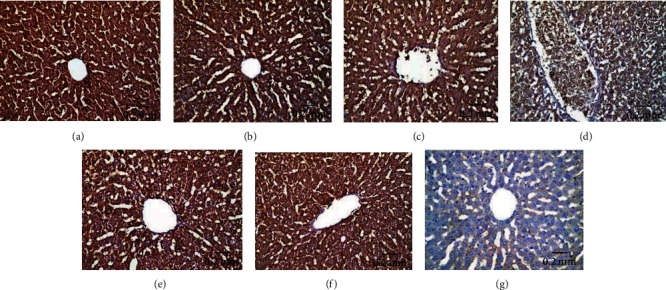
Immunohistochemical expression of Bcl-2 in hepatic tissues illustrating (a and b) strong reaction in control and Eug-treated group, respectively; (c) moderate reactivity in the AgNPs-low-dose-treated group; (d) weak immunostainability in the AgNPs-high-dose-treated group; (e) strong immunostaining in cotreatment of Eug with AgNPs-low-dose-treated group; (f) moderate immunostainability in Eug + AgNPs-high-dose-treated group; (g) no staining in negative control.

Bcl-2 immunoexpression in hepatic tissues of experimental animal groups is illustrated in Figures 11(a)–11(g). AgNPs-low-dose-treated rats showed moderate Bcl-2 immunoreactivity (Figure 11(c)), whereas AgNPs-high-dose-treated rats revealed weak Bcl-2 immunoreactivity (Figure 11(d)) compared with the control (Figure 11(a)) and Eug-treated rat (Figure 11(b)) groups which showed strong Bcl-2 immunoreactivity. However, treatment of rats with Eug paralleled with AgNPs upregulated the immunoexpression of Bcl-2 in both the AgNPs-low-dose (Figure 11(e)) and AgNPs-high-dose (Figure 11(f)) groups. Negative control sample revealed negative immunoreactivity (Figure 11(g)). As shown in Table 3, Eug-treated rats did not exhibit significant change (*P* > 0.05) in the value of the area percentage of Bcl-2 immunoexpression in comparable with that of control rats. Meanwhile, a significant decline (*P* ≤ 0.05) in the area percentage of Bcl-2 immunoexpression was recorded in the AgNPs-low-dose- and AgNPs-high-dose-treated groups, which was significantly (*P* ≤ 0.05) modulated in the rats cotreated with Eug.

#### 3.5.2. P53 Immunoreactivity

**Figure 12:**
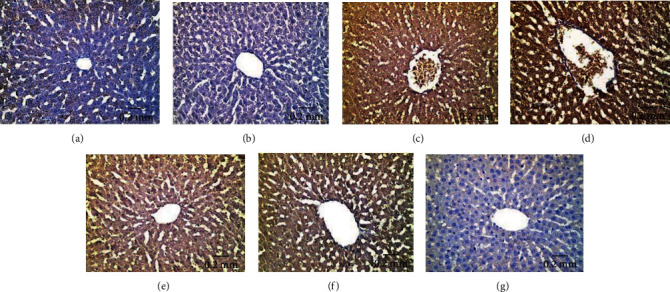
Immunohistochemical expression of P53 in hepatic tissues revealing (a and b) weak immunostainability in the control and Eug-treated groups, respectively; (c) moderate positive immunostainability in the AgNPs-low-dose-treated group; (d) relatively strong positive immunoreaction in the AgNPs-high-dose-treated group; (e) weak positive affinity for P53 in the Eug + ANPs-low-dose-treated group, (f) moderate immunoreactivity in the Eug + ANPs-high-dose-treated group; (g) negative immunostaining in negative control.

Hepatic tissues of control (Figure 12(a)) and Eug-treated (Figure 12(b)) rats revealed weak P53 immunoreactivity, whereas the rats treated with AgNPs-low dose manifested a moderate P53 immunoreactivity (Figure 12(c)). Furthermore, AgNPs-high-dose-treated rats exhibited intense positive stainability for P53 (Figure 12(d)). Meanwhile, Eug + AgNPs-low-dose-treated rats showed mild P53 immunoreactivity (Figure 12(e)), whereas Eug + AgNPs-high-dose-treated rats illustrated moderate reaction for P53 immunostaining (Figure 12(f)). There was no staining in negative control samples (Figure 12(g)). As revealed in Table 3, Eug-treated rats did not reveal any significant difference (*P* > 0.05) in the area percentage of P53 immunoexpression compared with the value of control group, whereas a significant elevation (*P* ≤ 0.05) in the area percentage of P53 immunoexpression was recorded in both AgNPs-low-dose and AgNPs-high-dose-treated groups. However, a significant rise (*P* ≤ 0.05) in the area percentage of P53 immunoexpression was recorded for both Eug + AgNPs-low-dose- and Eug + AgNPs-high-dose-treated rats relative to the control group.

#### 3.5.3. Caspase-3 Immunoreactivity

**Figure 13:**
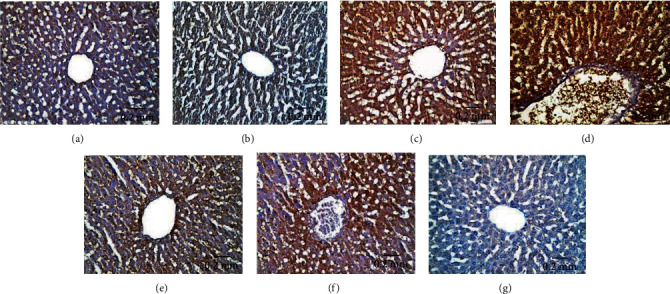
Immunohistochemical expression of Caspase-3 in hepatic tissues showing (a and b) a weak immunostainability in the control and Eug-treated groups, respectively, (c) moderate immunoreaction in the AgNPs-low-dose-treated group, (d) strong positive immunostainability in AgNPs-high-dose-treated group, (e and f) moderate immunoreaction in both Eug + AgNPs-low- and high-dose-treated groups, (g) no stainability in negative control.

Immunohistochemical examination of hepatic tissues of the control (Figure 13(a)) and Eug-treated (Figure 13(b)) groups exhibited weak Caspase-3 immunostainability, while liver sections of AgNPs-low-dose-treated rats showed strong positive Caspase-3 immunoreactivity (Figure 13(c)). Hepatic tissues of AgNPs-high-dose-treated rats manifested an intense immunostainability for Caspase-3 (Figure 13(d)). Eug coadministered with both AgNPs-low-dose- and AgNPs-high-dose-treated groups illustrated moderate Caspase-3 immunoreactivity (Figures 13(e) and 13(f)), respectively. No staining was observed in negative control section (Figure 13(g)). According to Table 3, Eug-treated rats did not show a significant increase (*P* > 0.05) in the area percentage of Caspase-3 immunoexpression relative to control rats, while low-dose and high-dose AgNPs-treated rats evoked a significant increase (*P* ≤ 0.05) in the area percentage of Caspase-3 immunoexpression in comparable with control animals. Concomitant administration of Eug to the rats treated with low and high doses of AgNPs caused modulation of the area percentage of Caspase-3 immunoexpression in contrast with the AgNPs-low- or high-dose-treated groups but still showed a significant elevation (*P* ≤ 0.05) compared with the control group.

#### 3.5.4. TNF-*α* Immunoreactivity

**Figure 14:**
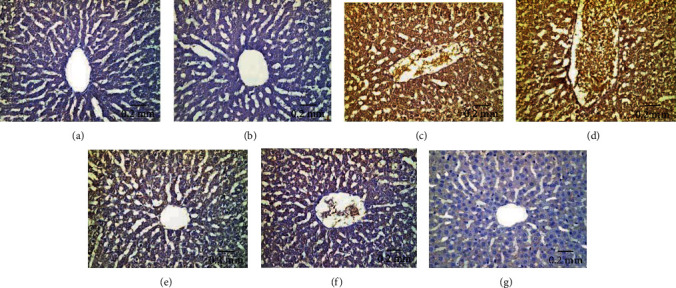
Immunohistochemical expression of TNF-*α* in hepatic tissues showing (a and b) negative immunohistochemical reactivity in control and Eug-treated rats, respectively; (c) moderate immunostainability in the AgNPs-low-dose-treated group; (d) strong immunostainability in the AgNPs-high-dose-treated group; (e) mild immunostainability in the Eug + AgNPs-low-dose-treated group; (f) weak immunoreactivity in the Eug + AgNPs-high-dose-treated group; (g) no immunostainability in negative control.

The liver sections of the control (Figure 14(a)) and Eug-treated (Figure 14(b)) groups showed negative TNF-*α* immunohistochemical reactivity, whereas the liver sections of AgNPs-low-dose-treated rats (Figure 14(c)) and AgNPs-high-dose-treated rats (Figure 14(d)) showed strong positive TNF-*α* immunostainability. Meanwhile, the hepatic tissues of Eug coadministered with the AgNPs-low-dose- (Figure 14(e)) or AgNPs-high-dose-treated (Figure 14(f)) groups illustrated mild TNF-*α* immunoreactivity compared with rats treated with low or high dose of AgNPs alone.  Figure 14(g) shows no immunoexpression in negative control sample. As revealed in Table 3, Eug-treated rats did not exhibit significant (*P* > 0.05) difference in the area percentage of TNF-*α* immunoexpression compared to control rats, while low- and high-dose AgNPs-treated rats evoked a significant rise (*P* ≤ 0.05) in the area percentage of TNF-*α* immunoexpression compared with those of control group. However, accompanying administration of Eug to low- or high-dose AgNPs-treated rats showed amendment of TNF-*α* immunoexpression compared with the rats subjected to AgNPs low or high doses alone.

## 4. Discussion

The usage of NPs is becoming increasingly popular in various disciplines. Due to their minuscule size and unique chemical and physical characteristics, AgNPs have become one of the most commercialized NPs worldwide. While the use of AgNPs for biological applications is increasing, our knowledge and understanding of how they affect living cells and biochemical structures is still lacking [45]. Therefore, the potential toxicity of AgNPs in different body organs is an important research area. Regarding AgNPs toxicity, there are no published studies on the ameliorating role of Eug against the toxic influences of AgNPs.

Among varied organs, the liver is the principal objective organ of AgNPs' effects via all exposure pathways [2, 46, 47]. Thus, the current investigation was aimed to prepare and characterize AgNPs and to evaluate their toxicity on the biochemical, histological, histomorphometrical, and immunohistochemical characteristics of the hepatic tissues of adult rats. Furthermore, the ameliorative role of Eug in the hepatic structure and function against AgNPs-induced hepatotoxicity was investigated.

The present results revealed that the microwave technique reduces AgNPs with variable-rate microwave radiation. The microwave technique speeds up chemical reactions from days or hours to minutes. With an absorbance peak at 413 nm, microwave irradiation allows homogeneous heating for NPs formation and aids in the ripening of these nanomaterials without agglomeration [48]. The sample synthesized at 413 nm was used to trigger hepatotoxicity [2], and it facilitated AgNPs passage into the hepatocytes.

The current study revealed that the applied doses of AgNPs significantly increased serum AST, ALT, ALP, and LDH levels, which are the most indicative markers of structural deterioration of hepatocytes because these enzymes are located in the cytoplasm and released into the blood due to the loss of functional membrane integrity and cellular leakage [49]. Additionally, AgNPs induced a significant drop in serum total protein and albumin levels, indicating a decrease in protein synthesis and/or an increase in protein catabolism [50]. Our findings were consistent with previous studies [51], which showed that an AgNPs overdose might cause liver deterioration. Administration of Eug alleviated AgNPs-induced hepatotoxicity, as indicated by significant decline in AST, ALT, ALP, and LDH levels and marked elevation of total protein and total albumin levels, revealing the preservation of the functional integrity of hepatocytes. These findings were consistent with those previously published by [52], who found that cotreatment of Eug with cadmium considerably improves AST, ALT, ALP, and LDH levels in rats. Eug has antihepatotoxic activity, making it a suitable dietary supplement for treating hepatic damage [53].

Free radicals are extremely reactive molecules that originate as ordinary byproducts of metabolic activities in living cells. These chemicals include reactive nitrogen (RNS) and oxygen (ROS) species which can speedily interact with different macromolecules (proteins, carbohydrates, lipids, and nucleic acids) inside the cells, significantly damaging cellular structures [54]. Living cells produce endogenous antioxidative factors to buffer such synthesized free radicals, protecting cells from oxidative harm. CAT, SOD, GPx, and GSH are the most prevalent endogenous antioxidant molecules [55]. When the produced free radicals exceed the cell's ability to countervail them with antioxidant molecules, oxidative stress occurs [56]. MDA is also a key indicator of oxidative stress in biological systems, and it is formed by ROS-induced peroxidation of membrane lipids, resulting in membrane damage and degradation [57].

The increased serum TOC level and the decreased TAC level, along with significantly increased LPO levels, reduced GSH levels, and decreased efficacy of CAT, SOD, and GPx in hepatic tissues of rats administered AgNPs, suggest that LPO is enhanced, causing excess free radical production, which triggers a chain interaction of direct and indirect bond formation with cellular molecules, impairing pivotal cellular activities and potentially leading to significant cell destruction and death [58]. The mitochondria are essential cellular targets of AgNPs-induced toxicity because AgNPs affect mitochondrial membrane permeability and interfere with their respiratory chain, resulting in ROS generation, necrosis, and apoptosis [59]. Previous studies have also revealed oxidative stress following AgNPs administration, which is consistent with our findings [2, 46, 60]. However, cotreatment of Eug with AgNPs induces the protective influence of Eug against the AgNPs-induced adverse alterations in oxidative stress biomarkers. Eug prevents LPO by disrupting the chain reaction by trapping active oxygen and being metabolized to a dimer (dieugenol), which prevents LPO at the level of free radical chain reaction propagation [61].

TNF-*α* and IL-6 are proinflammatory cytokines that are involved in immunological retrogradation by mediating tissue inflammation and organ injury [62]. Rats intoxicated with AgNPs showed significantly elevated hepatic TNF-*α* and IL-6 levels, indicating that AgNPs primarily influence macrophage activities and promote liver injury development. Our findings are consistent with earlier researches that found inflammation in the rat's liver after AgNPs treatment [52, 63]. Nuclear factor-kappa B (NF-*κ*B) is a protein complex required for activating the inflammatory cascade that causes IL-6 and TNF-*α* transcription [64]. Inflammasomes are multiprotein complexes that initiate inflammatory reactivity [65]. AgNPs activate both NF-*κ*B transcriptional and inflammasome paths, indicating the participation of these paths in the molecular mechanism underlying AgNPs' inflammatory effects [66]. The current results revealed marked suppression of the overproduction of the tested cytokines in the liver tissues of rats treated with Eug concomitant with AgNPs-low or AgNPs-high doses. Eug has long been recognized for its anti-inflammatory properties, since it inhibits varied inflammation-related signaling routes containing the NF-*κ*B pathway [52].

The present biochemical outcomes were proved by our histological, histomorphometrical, and immunohistochemical observations. Histopathologically, varied alterations reflecting the hepatotoxic effects of AgNPs, included hepatocellular degeneration, lymphocytic infiltration, hepatocytic necrosis/apoptosis, dilatation/congestion of blood vessels, and dilation of sinusoidal spaces were the most identified hepatic abnormalities. In addition, the two applied doses of AgNPs significantly increased the numbers of necrotic hepatocytes, infiltrating inflammatory cells, deteriorated Kupffer cells, dilated/congested central and portal veins, besides significantly decreasing the numbers of normal hepatic cells, which are the most indicative signs of hepatic structural damage. Furthermore, the pathological responses of hepatic tissues to AgNPs were dose dependent, since the high AgNPs dose was more efficient compared with the low dose.

The majority of hepatocytes of AgNPs-treated rats manifested severe degeneration distinguished by clear signs of necrosis. Such degradation could be associated with leakage of lysosomal hydrolytic enzymes, as well as disruption of hepatocyte membrane function, which causes a huge influx of water and Na+, leading to cytoplasmic degeneration [67]. Furthermore, necrosis is triggered by an attack on the cell organelles, particularly the endoplasmic reticulum, mitochondria, and nucleus, which disrupts their functions [68]. In this study, necrosis was evidenced by GSH depletion and diminishing in CAT and SOD activities [2].

The current study's findings manifested that vascular congestion was virtually a constant hallmark in hepatic tissues of AgNPs-treated rats, where some of these blood vessels had distorted contours owing to the surrounding fibrosis. Blood extravasation was also seen. Sinusoidal dilatation has also been elucidated as a result of hepatocyte necrosis or injury of the lining sinusoidal endothelial tissue [69]. Furthermore, the prominence and swelling of Kupffer cells were among the most noticeable AgNPs toxicity. These lesions are ascribed to the vital role of Kupffer cells in bodily protective mechanism versus detoxification of AgNPs-induced hepatic oxidative stress, because Kupffer cells are the first cells exposed to the hazardous compounds entering the liver via the portal vein [47, 70].

The hepatic tissues of AgNPs-treated rats showed dispersed inflammatory cell infiltration in the centrilobular and periportal zones, indicating that AgNPs interact with the interstitial hepatic tissues, causing a variety of immunological responses [71]. Comparable results were reported by [2], who confirmed that leukocytosis is found in AgNPs-treated animals.

The present immunohistochemical findings showed that AgNPs-mediated cell death is connected with apoptosis, which was confirmed by significant decline in Bcl-2 immunoreactivity and elevation in P53 and Caspase-3 immunoreactivities after low- and high-dose AgNPs treatment. Bcl-2, an antiapoptotic protein that is overexpressed in many cancers, inhibits the intrinsic mitochondria-mediated cell death process by preventing mitochondrial membrane permeabilization, which causes proapoptotic chemical leakage [72]. Moreover, Bcl-2 gene family controls caspase activation in the intrinsic apoptosis pathway, which is induced by intracellular harm, such as DNA damage [73]. The activation of P53 is linked to apoptosis induction and also regulates the expression of other apoptosis-related proteins [74]. [75] believed that the stimulation of apoptosis and alteration of gene expression of the apoptosis-associated genes Bcl-2 and P53 is responsible for cancer cell death induced by biogenic metal NPs. Caspase-3 is an apoptotic marker that can be triggered by both intrinsic and extrinsic apoptotic pathways, resulting in DNA breakage [76]. These changes can be attributed to AgNPs aggregation in tissues [77] or to the AgNPs' effect on mitochondrial activity, reducing cell viability [46].

Intracellular AgNPs toxicity can be brought on by different processes, especially excessive ROS generation. AgNPs possess unique characteristics that enable them to penetrate cell membranes and other biological barriers, causing cellular malfunction or destruction [47]. Oxidative stress increases intracellular ROS, as confirmed by our biochemical results. Metal oxide NPs trigger DNA damage and cell death through ROS generation and oxidative damage [78]. ROS can trigger cell death via two separate cell death mechanisms, apoptosis and necrosis. ROS also activate caspases, which are believed to execute apoptosis. These modifications can eventually lead to organ malfunction or even cancer [79]. Since biochemical decrease in GPx, CAT, and SOD essentially results in oxidative stress-induced cell death, such apoptotic effects could potentially be a consequence of the AgNPs-induced intercellular oxidative stress [2]. [80] illustrated that nanochelating-based AgNPs cause mild apoptosis/necrosis in mice, as well as changes in numerous clinical variables, such as blood parameters and liver enzymes.

Although TNF-*α* is released from primary hepatocytes, it is implicated in the proinflammatory response and cell-to-cell communication, and its signaling is linked to many autoimmune and inflammatory diseases. TNF-*α* is primarily formed by macrophages in inflammatory tissues, and it is involved in angiogenesis, wound healing, and tumor formation [81]. In this study, TNF-*α* expression was not detected in normal liver specimens but was overexpressed in liver tissues of AgNPs-treated rats. Our findings are in line with those of other researchers who found higher TNF-*α* production in chronic liver disease [82].

On the other side, coadministration of Eug alongside AgNPs attenuated the intensity of the histopathological and histomorphometrical changes in the hepatic tissues of rats treated with low and high doses of AgNPs. Also, concomitant treatment with Eug into rats exposed to AgNPs modulated the immunohistochemical expression of Bcl-2, P53, Caspase-3, and TNF-*α* reactive proteins. These results are consistent with those reported by [52, 53, 61]. The hepatoprotective efficiency of Eug can be attributed to its ability to stabilize hepatocyte membranes by preventing LPO and improving antioxidant enzyme activity, besides its free radical scavenging and anti-inflammatory properties.

## 5. Conclusions

The current investigation established that exposing rats to AgNPs caused oxidative stress and inflammation, which resulted in hepatotoxicity. Our results also provide a new insight into the ameliorative role of Eug supplementation against AgNPs-induced hepatotoxicity due to its antioxidant, antiapoptotic, and anti-inflammatory properties. Finally, we suggest using Eug as a preventive agent along with AgNPs to minimize its hepatotoxicity.

## Figures and Tables

**Table 1:** The utilized antibodies in the immunohistochemical investigation.

Antibody	Code	Clone	Antigen retrieval	Dilution	Sources	Company
Bcl-2	MA5-11757	100/D5	PBS, pH 7.4 with 0.2% BSA	1 : 50	Mouse/IgG1, kappa	Thermo Fisher Scientific (USA)
P53	MA5-12557	DO-7	PBS, pH 7.4	1 : 100-1 : 200	Mouse/IgG2b, kappa	Thermo Fisher Scientific
Caspase-3	MA5-11516	3CSP01 (7.1.44)	PBS, pH 7.4, with 0.2% BSA	1 : 50-1 : 100	Mouse/IgG2a	Thermo Fisher Scientific
TNF-*α*	MA5-23720	28401	PBS with 5% trehalose	8-25 *μ*g/mL	Mouse/IgG1	Thermo Fisher Scientific

**Table 2:** Histomorphometrical analysis of the hepatic tissue components in control and treated animal groups.

Parameters	Animal groups					
	Control	Eug	AgNPs-low dose	AgNPs-high dose	Eug + AgNPs-low dose	Eug + AgNPs-high dose
Number of normal hepatocytes	316 ± 15.3^a^	300 ± 0^a^	260 ± 0^b^	176 ± 0.775^c^	290 ± 0^a^	240 ± 0^b^
% change	0	-5.06%	-17.72%	-44.30%	-8.23%	-24.05%

Number of necrotic hepatocytes	5.2 ± 0^f^	7.2 ± 0^e^	20.2 ± 0^c^	63.8 ± 0^a^	12.6 ± 0^d^	21.6 ± 0^b^
% change	0	38.46%	288.46%	1126.92%	142.31%	315.38%

Number of deteriorated Kupffer cells	12 ± 0.316^d^	14 ± 0^d^	23.4 ± 0^c^	58 ± 2.47^a^	16.2 ± 0^d^	28.6 ± 0^b^
% change	0	16.67%	95.00%	383.33%	35.00%	138.33%

Number of periportal inflammatory cells	10.2 ± 0^f^	12.6 ± 0^e^	35 ± 0^c^	80.6 ± 0^a^	18.2 ± 0^d^	40.4 ± 0^b^
% change	0	23.53%	243.14%	690.20%	78.43%	296.08%

Number of centrilobular inflammatory cells	6.8 ± 0^d^	8.4 ± 0.6^d^	20.4 ± 0^c^	41.6 ± 3.89^a^	12.8 ± 0^d^	28.6 ± 0^b^
% change	0	23.53%	200.00%	511.76%	88.24%	320.59%

Number of dilated/congested central veins	0.4 ± 0.245^c^	0.6 ± 0.245^c^	2 ± 0^b^	4.8 ± 0^a^	1 ± 0^c^	1.8 ± 0^b^
% change	0	50.00%	400.00%	1100.00%	150.00%	200.00%

Number of dilated/congested portal veins	0.6 ± 0.245^c^	0.8 ± 0.2^c^	2 ± 0^b^	4.6 ± 0.245^a^	1.2 ± 0^c^	2.4 ± 0^b^
% change	0	33.33%	233.33%	666.67%	100.00%	200.00%

Data are presented as Mean ± SEM and % change (*n* = 6 in each group). Within the same row, values preceded by different superscript letters differ significantly at the 0.05 level (one-way ANOVA and the Tukey test). Eug: eugenol; AgNPs: silver nanoparticles.

**Table 3:** Immunohistochemical image analysis of the area percentage of Bcl-2, P53, Caspase-3, and TNF-*α* immunoexpression in hepatic tissues of control and treated animal groups.

Parameters	Animal groups					
	Control	Eug	AgNPs-low dose	AgNPs-high dose	Eug + AgNPs-low dose	Eug + AgNPs-high dose
Bcl-2	67.8 ± 1.78^a^	65.1 ± 3.76^a^	55.6 ± 0^bc^	42.1 ± 0.51^d^	62.4 ± 0^ab^	53.6 ± 0^c^
% change	0	-3.98%	-17.99%	-37.91%	-7.96%	-20.94%

P53	20.8 ± 2.27^c^	25.8 ± 0.58^c^	42.8 ± 4.12^b^	63 ± 3.66^a^	28.2 ± 0^c^	45.5 ± 0^b^
% change	0	24.04%	105.77%	202.88%	35.58%	118.75%

Caspase-3	20.74 ± 2.06^c^	26 ± 0^c^	40.4 ± 0^b^	67.57 ± 2.19^a^	29 ± 0^c^	43 ± 0^b^
% change	0	25.31%	94.71%	225.64%	39.77%	107.24%

TNF-*α*	22 ± 2.97^c^	26.6 ± 0.75^c^	39.8 ± 2.44^b^	68.6 ± 4.23^a^	28.6 ± 0^c^	40.1 ± 0^b^
% change	0	20.91%	80.91%	211.82%	30.00%	82.27%

Data are presented as Mean ± SEM and % change (*n* = 6 in each group). Within the same row, values preceded by different superscript letters differ significantly at the 0.05 level (one-way ANOVA and the Tukey test). Eug: eugenol; AgNPs: silver nanoparticles.

## Data Availability

The data presented in this study are available on request from the corresponding author.

## References

[1] Singh R., Nalwa H. S. (2011). Medical applications of nanoparticles in biological imaging, cell labeling, antimicrobial agents, and anticancer nanodrugs. *Journal of Biomedical Nanotechnology*.

[2] El Mahdy M. M., Eldin T. A. S., Aly H. S., Mohammed F. F., Shaalan M. I. (2015). Evaluation of hepatotoxic and genotoxic potential of silver nanoparticles in albino rats. *Experimental and Toxicologic Pathology*.

[3] Nedelcu I. A., Ficai A., Sonmez M., Ficai D., Oprea O., Andronescu E. (2014). Silver based materials for biomedical applications. *Current Organic Chemistry*.

[4] Kraeling M. E. K., Topping V. D., Keltner Z. M. (2018). _In vitro_ percutaneous penetration of silver nanoparticles in pig and human skin. *Regulatory Toxicology and Pharmacology*.

[5] Kumar S., Shukla A., Baul P. P., Mitra A., Halder D. (2018). Biodegradable hybrid nanocomposites of chitosan/gelatin and silver nanoparticles for active food packaging applications. *Food Packaging and Shelf Life*.

[6] Zhou Y., Tang R.-C. (2018). Facile and eco-friendly fabrication of AgNPs coated silk for antibacterial and antioxidant textiles using honeysuckle extract. *Journal of Photochemistry and Photobiology B: Biology*.

[7] Dankovich T. A., Gray D. G. (2011). Bactericidal paper impregnated with silver nanoparticles for point-of-use water treatment. *Environmental Science & Technology*.

[8] Lee S. J., Morrill A. R., Moskovits M. (2006). Hot spots in silver nanowire bundles for surface-enhanced Raman spectroscopy. *Journal of the American Chemical Society*.

[9] Park W., Na K. (2015). Advances in the synthesis and application of nanoparticles for drug delivery. *WIREs Nanomedicine and Nanobiotechnology*.

[10] Ferdous Z., Nemmar A. (2020). Health impact of silver nanoparticles: a review of the biodistribution and toxicity following various routes of exposure. *International Journal of Molecular Sciences*.

[11] Gaiser B. K., Hirn S., Kermanizadeh A. (2013). Effects of silver nanoparticles on the liver and hepatocytes in vitro. *Toxicological Sciences*.

[12] Patlolla A. K., Hackett D., Tchounwou P. B. (2015). Silver nanoparticle-induced oxidative stress-dependent toxicity in Sprague-Dawley rats. *Molecular and Cellular Biochemistry*.

[13] McShan D., Ray P. C., Yu H. (2014). Molecular toxicity mechanism of nanosilver. *Journal of Food and Drug Analysis*.

[14] Forni C., Facchiano F., Bartoli M. (2019). Beneficial role of phytochemicals on oxidative stress and age-related diseases. *BioMed Research International*.

[15] Khalil A. A., Rahman U. ., Khan M. R., Sahar A., Mehmood T., Khan M. (2017). Essential oil eugenol: sources, extraction techniques and nutraceutical perspectives. *RSC Advances*.

[16] Mishra A. K., Mishra A., Kehri H. K., Sharma B., Pandey A. K. (2009). Inhibitory activity of Indian spice plant Cinnamomum zeylanicum extracts against Alternaria solani and Curvularia lunata, the pathogenic dematiaceous moulds. *Annals of Clinical Microbiology and Antimicrobials*.

[17] Murcia M. A., Egea I., Romojaro F., Parras P., Jiménez A. M., Martínez-Tomé M. (2004). Antioxidant evaluation in dessert spices compared with common food additives. Influence of irradiation procedure. *Journal of Agricultural and Food Chemistry*.

[18] Tung Y. T., Chua M. T., Wang S. Y., Chang S. T. (2008). Anti-inflammation activities of essential oil and its constituents from indigenous cinnamon (Cinnamomum osmophloeum) twigs. *Bioresource Technology*.

[19] Dervis E., Yurt Kilcar A., Medine E. I. (2017). In vitro incorporation of radioiodinated eugenol on adenocarcinoma cell lines (Caco2, MCF7, and PC3). *Cancer Biotherapy & Radiopharmaceuticals*.

[20] Zahran H. Y., Kilany M., Yahia I. S., Albulym O., Hussien M. S. A., Abutalib M. M. (2019). Facile microwave synthesis of silver nanoplates: optical plasmonic and antimicrobial activity. *Materials Research Express*.

[21] Zhang X. F., Liu Z. G., Shen W., Gurunathan S. (2016). Silver nanoparticles: synthesis, characterization, properties, applications, and therapeutic approaches. *International Journal of Molecular Sciences*.

[22] Dykstra M. J., Reuss L. E. (2003). *Biological Electron Microscopy: Theory, Techniques, and Troubleshooting*.

[23] Reddy A. C., Lokesh B. R. (1996). Effect of curcumin and eugenol on iron-induced hepatic toxicity in rats. *Toxicology*.

[24] Lowry O. H., Rosebrough N. J., Farr A. L., Randall R. J. (1951). Protein measurement with the Folin phenol reagent. *Journal of Biological Chemistry*.

[25] Tietz N. W. (1976). *Fundamentals of Clinical Chemistry*.

[26] Vassault A., Azzedine M. C., Bailly M. (1986). Protocole de validation de techniques. Commission Validation de techniques. *Annales de Biologie Clinique*.

[27] Armstrong W., Carr C. (1964). Estimation of serum total protein. *Physiological Chemistry Laboratory Directions*.

[28] Doumas B. T., Ard Watson W., Biggs H. G. (1971). Albumin standards and the measurement of serum albumin with bromcresol green. *Clinica Chimica Acta*.

[29] Koracevic D., Koracevic G., Djordjevic V., Andrejevic S., Cosic V. (2001). Method for the measurement of antioxidant activity in human fluids. *Journal of Clinical Pathology*.

[30] Tatzber F., Griebenow S., Wonisch W., Winkler R. (2003). Dual method for the determination of peroxidase activity and total peroxides- iodide leads to a significant increase of peroxidase activity in human sera. *Analytical Biochemistry*.

[31] Buege J. A., Aust S. D. (1978). Microsomal lipid peroxidation. *Methods in Enzymology*.

[32] Nishikimi 7M., Appaji Rao N., Yagi K. (1972). The occurrence of superoxide anion in the reaction of reduced phenazine methosulfate and molecular oxygen. *Biochemical and Biophysical Research Communications*.

[33] Aebi H. (1984). Catalase in vitro. *Methods in Enzymology*.

[34] Beutler E., Duron O., Kelly B. M. (1963). Improved method for the determination of blood glutathione. *The Journal of Laboratory and Clinical Medicine*.

[35] Paglia D. E., Valentine W. N. (1967). Studies on the quantitative and qualitative characterization of erythrocyte glutathione peroxidase. *The Journal of Laboratory and Clinical Medicine*.

[36] Bancroft J. D., Gamble M. (2008). Theory and Practice of Histological Techniques.

[37] Petrosyan K., Tamayo R., Joseph D. (2002). Sensitivity of a novel biotin-free detection reagent (Powervision+<SUP>™</SUP>) for immunohistochemistry. *Journal of Histotechnology*.

[38] Van Eycke Y. R., Allard J., Salmon I., Debeir O., Decaestecker C. (2017). Image processing in digital pathology: an opportunity to solve inter-batch variability of immunohistochemical staining. *Scientific Reports*.

[39] Mofolo M. J., Kadhila P., Chinsembu K. C., Mashele S., Sekhoacha M. (2020). Green synthesis of silver nanoparticles from extracts of Pechuel-loeschea leubnitziae: their anti-proliferative activity against the U87 cell line. *Inorganic and Nano-Metal Chemistry*.

[40] Liu Z., Wang L., Zhao X., Luo Y., Zheng K., Wu M. (2022). Highly effective antibacterial [email protected] grafted chitosan for construction of durable antibacterial fabrics. *International Journal of Biological Macromolecules*.

[41] Ghodake G., Shinde S., Saratale R. G. (2019). Whey peptide-encapsulated silver nanoparticles as a colorimetric and spectrophotometric probe for palladium(II). *Microchimica Acta*.

[42] Bhuyar P., Rahim M. H. A., Sundararaju S., Ramaraj R., Maniam G. P., Govindan N. (2020). Synthesis of silver nanoparticles using marine macroalgae Padina sp. and its antibacterial activity towards pathogenic bacteria. *Beni-Suef University Journal of Basic and Applied Sciences*.

[43] Shah Z., Gul T., Ali Khan S. (2021). Synthesis of high surface area AgNPs from _Dodonaea viscosa_ plant for the removal of pathogenic microbes and persistent organic pollutants. *Materials Science and Engineering: B*.

[44] Jyoti K., Baunthiyal M., Singh A. (2016). Characterization of silver nanoparticles synthesized using Urtica dioica Linn. leaves and their synergistic effects with antibiotics. *Journal of Radiation Research and Applied Sciences*.

[45] Islam M. A., Jacob M. V., Antunes E. (2021). A critical review on silver nanoparticles: from synthesis and applications to its mitigation through low-cost adsorption by biochar. *Journal of Environmental Management*.

[46] Hussain S. M., Hess K. L., Gearhart J. M., Geiss K. T., Schlager J. J. (2005). In vitro toxicity of nanoparticles in BRL 3A rat liver cells. *Toxicology In Vitro*.

[47] Pourhamzeh M., Gholami Mahmoudian Z., Saidijam M., Asari M. J., Alizadeh Z. (2016). The effect of silver nanoparticles on the biochemical parameters of liver function in serum, and the expression of Caspase-3 in the liver tissues of male rats. *Avicenna Journal of Medical Biochemistry*.

[48] Pal J., Deb M. K., Deshmukh D. K. (2014). Microwave-assisted synthesis of silver nanoparticles using benzo-18-crown-6 as reducing and stabilizing agent. *Applied Nanoscience*.

[49] Chaung S. S., Lin C. C., Lin J., Yu K. H., Hsu Y. F., Yen M. H. (2003). The hepatoprotective effects of Limonium sinense against carbon tetrachloride and *β*-D-galactosamine intoxication in rats. *Phytotherapy Research*.

[50] Abdel-Wahhab M. A., Aly S. E. (2005). Antioxidant property of Nigella sativa (black cumin) and Syzygium aromaticum (clove) in rats during aflatoxicosis. *Journal of Applied Toxicology*.

[51] Mosa I. F., Youssef M., Shalaby T., Mosa O. F. (2019). The protective role of tannic acid against possible hepato-nephrotoxicity induced by silver nanoparticles on male rats. *Sanamed*.

[52] Kumar A., Siddiqi N. J., Alrashood S. T., Khan H. A., Dubey A., Sharma B. (2021). Protective effect of eugenol on hepatic inflammation and oxidative stress induced by cadmium in male rats. *Biomedicine & Pharmacotherapy*.

[53] Marchese A., Barbieri R., Coppo E. (2017). Antimicrobial activity of eugenol and essential oils containing eugenol: a mechanistic viewpoint. *Critical Reviews in Microbiology*.

[54] Brieger K., Schiavone S., Miller F. J., Krause K. H. (2012). Reactive oxygen species: from health to disease. *Swiss Medical Weekly*.

[55] Aguilar T. A. F., Navarro B. C. H., Pérez J. A. M. (2016). *Endogenous Antioxidants: A Review of their Role in Oxidative Stress*.

[56] Ozcan A., Ogun M., Gowder S. J. T. (2015). *Biochemistry of Reactive Oxygen and Nitrogen Species, in Basic Principles and Clinical Significance of Oxidative Stress*.

[57] Busch C. J., Binder C. J. (2017). Malondialdehyde epitopes as mediators of sterile inflammation. *Biochimica et Biophysica Acta (BBA) - Molecular and Cell Biology of Lipids*.

[58] Halliwell B. (1994). Free radicals, antioxidants, and human disease: curiosity, cause, or consequence?. *Lancet*.

[59] Loghman A. (2012). Histopathologic and apoptotic effect of nanosilver in liver of broiler chickens. *African Journal of Biotechnology*.

[60] M Abd Elmaksoud E., M Taha N., A Lebda M., A Kamel M. (2019). The possible protective role of alpha-lipoic acid on nanosilver particle-induced hepatotoxicity in male rats. *Biochemistry Letters*.

[61] Ogata M., Hoshi M., Urano S., Endo T. (2000). Antioxidant activity of eugenol and related monomeric and dimeric compounds. *Chemical and Pharmaceutical Bulletin*.

[62] Lauwerys B. R., Houssiau F. A., Santamaria P., Hackett P. H. (2003). *Involvement of Cytokines in the Pathogenesis of Systemic Lupus Erythematosus, in Cytokines and Chemokines in Autoimmune Disease*.

[63] Ebabe Elle R., Gaillet S., Vidé J. (2013). Dietary exposure to silver nanoparticles in Sprague-Dawley rats: effects on oxidative stress and inflammation. *Food and Chemical Toxicology*.

[64] Shabab T., Khanabdali R., Moghadamtousi S. Z., Kadir H. A., Mohan G. (2017). Neuroinflammation pathways: a general review. *International Journal of Neuroscience*.

[65] Guo H., Callaway J. B., Ting J. P. (2015). Inflammasomes: mechanism of action, role in disease, and therapeutics. *Nature Medicine*.

[66] Fehaid A., Fujii R., Sato T., Taniguchi A. (2020). Silver nanoparticles affect the inflammatory response in a lung epithelial cell line. *The Open Biotechnology Journal*.

[67] Del Monte U. (2005). Swelling of hepatocytes injured by oxidative stress suggests pathological changes related to macromolecular crowding. *Medical Hypotheses*.

[68] Singh A., Bhat T. K., Sharma O. P. (2011). Clinical biochemistry of hepatotoxicity. *Journal of Clinical Toxicology*.

[69] Oligny L. L., Lough J. (1992). Hepatic sinusoidal ectasia. *Human Pathology*.

[70] Neyrinck A. (2004). Modulation of Kupffer cell activity: physio-pathological consequences on hepatic metabolism. *Bulletin et Mémoires de l'Académie Royale de Médecine de Belgique*.

[71] Johar D., Roth J. C., Bay G. H., Walker J. N., Kroczak T. J., Los M. (2004). Inflammatory response, reactive oxygen species, programmed (necrotic-like and apoptotic) cell death and cancer. *Roczniki Akademii Medycznej w Białymstoku*.

[72] Chan S. L., Yu V. C. (2004). Proteins of the bcl-2 family in apoptosis signalling: from mechanistic insights to therapeutic opportunities. *Clinical and Experimental Pharmacology & Physiology*.

[73] Cory S., Adams J. M. (2002). The Bcl2 family: regulators of the cellular life-or-death switch. *Nature Reviews Cancer*.

[74] May P., May E. (1999). Twenty years of p53 research: structural and functional aspects of the p53 protein. *Oncogene*.

[75] Banu H., Renuka N., Faheem S. M. (2018). Gold and silver nanoparticles biomimetically synthesized using date palm pollen extract-induce apoptosis and regulate p53 and Bcl-2 expression in human breast adenocarcinoma cells. *Biological Trace Element Research*.

[76] Ma W., Jing L., Valladares A. (2015). Silver nanoparticle exposure induced mitochondrial stress, Caspase-3 activation and cell death: amelioration by sodium selenite. *International Journal of Biological Sciences*.

[77] Sulaiman F. A., Adeyemi O. S., Akanji M. A. (2015). Biochemical and morphological alterations caused by silver nanoparticles in Wistar rats. *Journal of Acute Medicine*.

[78] Piao M. J., Kang K. A., Lee I. K. (2011). Silver nanoparticles induce oxidative cell damage in human liver cells through inhibition of reduced glutathione and induction of mitochondria-involved apoptosis. *Toxicology Letters*.

[79] Rahman K. (2007). Studies on free radicals, antioxidants, and co-factors. *Clinical Interventions in Aging*.

[80] Hoseini-Alfatemi S. M., Fallah F., Armin S., Hafizi M., Karimi A., Kalanaky S. (2020). Evaluation of blood and liver cytotoxicity and apoptosis-necrosis induced by nanochelating based silver nanoparticles in mouse model. *Iranian Journal of Pharmaceutical Research: IJPR*.

[81] Yang Y. M., Seki E. (2015). TNF*α* in liver fibrosis. *Current Pathobiology Reports*.

[82] McCaughan G. W., Gorrell M. D., Bishop G. A. (2000). Molecular pathogenesis of liver disease: an approach to hepatic inflammation, cirrhosis and liver transplant tolerance. *Immunological Reviews*.

